# Inflammatory Cytokine Elaboration Following Secondhand Smoke (SHS) Exposure Is Mediated in Part by RAGE Signaling

**DOI:** 10.3390/ijms242115645

**Published:** 2023-10-27

**Authors:** Katrina L. Curtis, Kyle M. Homer, Ryan A. Wendt, Brendan M. Stapley, Evan T. Clark, Kaden Harward, Ashley Chang, Derek M. Clarke, Juan A. Arroyo, Paul R. Reynolds

**Affiliations:** Lung and Placenta Laboratory, Department of Cell Biology and Physiology, Brigham Young University, Provo, UT 84602, USAryan.wendt00@gmail.com (R.A.W.); etclarkbyu@gmail.com (E.T.C.); ashemarkham@gmail.com (A.C.);

**Keywords:** RAGE, SHS, lung, chronic inflammation

## Abstract

The receptor for advanced glycation end products (RAGE) is a key contributor to immune and inflammatory responses in myriad diseases. RAGE is a transmembrane pattern recognition receptor with a special interest in pulmonary anomalies due to its naturally abundant pulmonary expression. Our previous studies demonstrated an inflammatory role for RAGE following acute 30-day exposure to secondhand smoke (SHS), wherein immune cell diapedesis and cytokine/chemokine secretion were accentuated in part via RAGE signaling. However, the chronic inflammatory mechanisms associated with RAGE have yet to be fully elucidated. In this study, we address the impact of long-term SHS exposure on RAGE signaling. RAGE knockout (RKO) and wild-type (WT) mice were exposed to SHS using a nose-only delivery system (Scireq Scientific, Montreal, Canada) for six months. SHS-exposed animals were compared to mice exposed to room air (RA) only. Immunoblotting was used to assess the phospho-AKT and phospho-ERK activation data, and colorimetric high-throughput assays were used to measure NF-kB. Ras activation was measured via ELISAs. Bronchoalveolar lavage fluid (BALF) cellularity was quantified, and a mouse cytokine antibody array was used to screen the secreted cytokines. The phospho-AKT level was decreased, while those of phospho-ERK, NF-kB, and Ras were elevated in both groups of SHS-exposed mice, with the RKO + SHS-exposed mice demonstrating significantly decreased levels of each intermediate compared to those of the WT + SHS-exposed mice. The BALF contained increased levels of diverse pro-inflammatory cytokines in the SHS-exposed WT mice, and diminished secretion was detected in the SHS-exposed RKO mice. These results validate the role for RAGE in the mediation of chronic pulmonary inflammatory responses and suggest ERK signaling as a likely pathway that perpetuates RAGE-dependent inflammation. Additional characterization of RAGE-mediated pulmonary responses to prolonged exposure will provide a valuable insight into the cellular mechanisms of lung diseases such as chronic obstructive pulmonary disease.

## 1. Introduction

Chronic obstructive pulmonary disease (COPD) describes an irreversible respiratory disorder characterized by chronic bronchitis and the emphysematous destruction of the lung parenchyma. Currently, there is no cure for COPD, and its treatment is limited to strategies that relieve the symptoms. Depending on the severity of the disease, the treatments can range from non-invasive measures, such as bronchodilators, steroids, antibiotics, or oxygen therapy, to invasive management, such as lung volume reduction surgery, lung transplants, or bullectomies [1]. Disease progression generally occurs due to long-term exposure to particulates found in combustible tobacco products or occupational exposure, but exacerbation is also associated with long-term exposure to air pollutants or fine biomass particulates [2]. Prolonged pulmonary inflammation, dysregulated protease production, and mucus hypersecretion contribute to alveolar destruction, decreased gas exchange, and an obstructed airflow [3]. As a result of such injuries, patients with COPD suffer from a composite of symptoms that typically include chronic dyspnea, fatigue, sputum, and a cough, all of which progressively worsen over time [4]. Many cases of COPD culminate in mortality; for instance, COPD was recently identified as the third leading cause of death worldwide, preceded only by ischemic heart disease and stroke [5]. 

While the major risk factor for developing COPD is primary smoking, studies estimate that approximately one quarter of all patients with COPD are not smokers [6,7,8,9]. One prominent risk factor associated with COPD rates in non-smokers is exposure to secondhand smoke (SHS) [2,10,11]. Although SHS exposure in the United States has declined in recent decades [12], exposure remains notable among children and adults that encounter ongoing passive smoke in public and at home. According to the 2019 National Youth Tobacco Survey, 25.3% of high school students in the United States reported exposure to SHS in the home, and another 23.3% reported SHS exposure in vehicles [13]. No amount of SHS exposure is considered to be risk-free, and chronic SHS exposure in both children and adults is associated with a higher risk of COPD mortality in adulthood [14]. 

Many cellular mechanisms and signaling pathways are involved in the pathology of COPD. One candidate progression factor in COPD pathogenesis is the receptor for advanced glycation end products (RAGE). RAGE is a transmembrane cell-surface protein of the immunoglobulin superfamily [15]. Instead of recognizing specific amino acid sequences in a ligands primary structure, the extracellular domain of the receptor binds to three-dimensional molecular entities, thereby classifying RAGE as a pattern recognition receptor (PRR) [16]. As a PRR, RAGE recognizes numerous ligands, including damage-associated molecular patterns (DAMPs), amyloid b peptides, S100 Calgranulins, and advanced glycation end products (AGEs) [17]. Of note, reactive glycation products are contained in cigarette smoke, and they function to rapidly cause AGE formation via Maillard chemical reactions both in vitro and in vivo [18]. Because no mechanism of degradation occurs during or after RAGE–ligand binding, the molecules can accumulate in an inflamed region and be delivered repeatedly to provide continual stimulation [19]. When activated, RAGE employs a host of cellular pathways to initiate signaling cascades that eventually contribute to the nuclear translocation of NF-kB. NF-kB transcriptionally regulates the gene programs of immune and inflammatory natures, including pro-inflammatory cytokines, chemokines, and cell adhesion molecules [20,21], as well as the upregulation of RAGE itself [22,23]. Thus, the activation of RAGE and its subsequent cellular pathways results in a state of prolonged inflammation that is perpetuated by continued stimuli. Because RAGE is expressed in a variety of cell types, including the epithelium, macrophages, motor neurons, vascular smooth muscle, and endothelial cells [24], it is implicated in several diseases, including inflammatory bowel and respiratory diseases, atherosclerosis, diabetes, and Alzheimer’s [25,26,27,28]. Despite such effects in various tissue types, the natural overexpression of RAGE in lung tissue particularly implicates it as a consequential factor in pulmonary diseases such as COPD [29]. 

Previous studies have indicated RAGE as a central perpetuator of acute pulmonary inflammatory responses to SHS [30,31,32]. The purpose of the current study was to expand previous research endeavors by investigating potential signaling pathways resulting from RAGE activation during long-term pulmonary inflammatory responses to SHS. A better understanding of such mechanisms may provide potential targets for future targeting therapeutics aimed at ameliorating the inflammatory exacerbations common among COPD patients. 

## 2. Results

### 2.1. Cellular Signaling Pathways Following Chronic SHS Exposure

**Figure 1:**
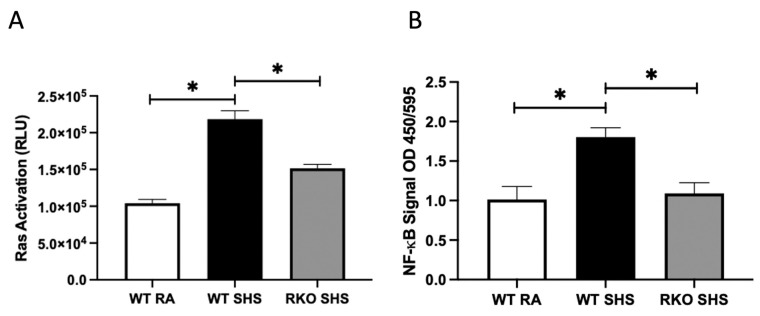
RAGE signaling related to SHS-mediated Ras and NF-kB expression. (**A**), Active Ras GTPase was more significantly increased in WT + SHS compared to that of WT + TA controls. Active Ras was also more significantly elevated in SHS-exposed RKO mice when compared to Ras activation in WT + SHS-exposed mice. Relative luciferase units (RLU) are reported. Data are representative of experiments (n = 6 mice per group), and significant differences are noted with * *p* ≤ 0.05. (**B**), Active NF-kB was more significantly elevated in WT + SHS-exposed mice compared to that of WT + RA controls and notably, NF-kB activation was more significantly diminished in RKO + SHS compared to that of WT+SHS-exposed mice. Data are representative of experiments (n = 6 mice per group), and significant differences are noted with * *p* ≤ 0.05. There was no difference in the expression of active Ras or NF-kB when comparing WT + RA and RKO + RA (not shown) controls.

**Figure 2:**
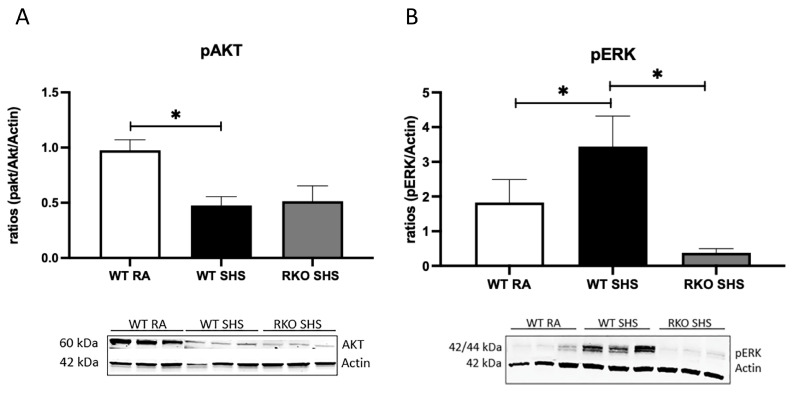
RAGE signaling related to SHS-mediated AKT and ERK expression. (**A**), Representative immunoblots for pAKT revealed significantly lower expression level in WT mice exposed to SHS compared to that of RA controls. Further, there was no significant difference observed between WT + SHS and RKO + SHS. (**B**), Differential expression of pERK was detected in representative immunoblots. Specifically, pERK level was more significantly increased in WT + SHS-exposed mice compared to that of WT + RA mice. Activated pERK level was significantly decreased in RKO + SHS-exposed mice when compared to pERK blots from RKO + SHS-exposed mice. Experiments were conducted in triplicate, and statistically different values are noted as * *p* ≤ 0.05. There was no difference in the expression of pAKT or pERK when comparing WT + RA and RKO + RA (not shown) controls.

Because RAGE expression following acute SHS exposure is known to perpetuate the inflammatory signaling pathways, we measured Ras activation, two key intracellular signaling intermediates (pAKT and pERK), and the downstream NF-kB levels in both the WT and RKO mice chronically exposed to RA or SHS. In a previous investigation, we revealed no significant differences in inflammatory responses when comparing WT mice and RKO mice following RA exposure only [33]. We discovered that after 6 months of exposure, the WT + SHS-exposed mice experienced the greater activation of Ras GTPase compared to that of the RA controls (Figure 1A). We also observed elevated Ras activation in the RKO mice exposed to SHS; interestingly, Ras activation was significantly attenuated in the RKO + SHS-exposed mice compared to that of the SHS-exposed WT mice (Figure 1A). Similarly, NF-kB activation was elevated in both the WT and RKO mice exposed to SHS, yet the RKO mice exposed to SHS expressed significantly less NF-kB activation compared to that of the SHS-exposed WT mice (Figure 1B). Immunoblotting revealed a significant decrease in pAKT activation in the lungs from the WT + SHS-exposed mice when compared to that of the WT + RA controls, and no significant change in pAKT expression was observed in the SHS-exposed RKO mice (Figure 2A). As AKT did not appear to be a central axis employed during RAGE signaling progression, ERK activation was observed to be partly mediated by RAGE availability. The ERK activation level was significantly increased in the WT + SHS-exposed mice, and that of pERK was significantly decreased in the RKO + SHS-exposed mice (Figure 2B).

### 2.2. BALF Cellularity and Cytokine Abundance Following Chronic SHS Exposure

**Figure 3:**
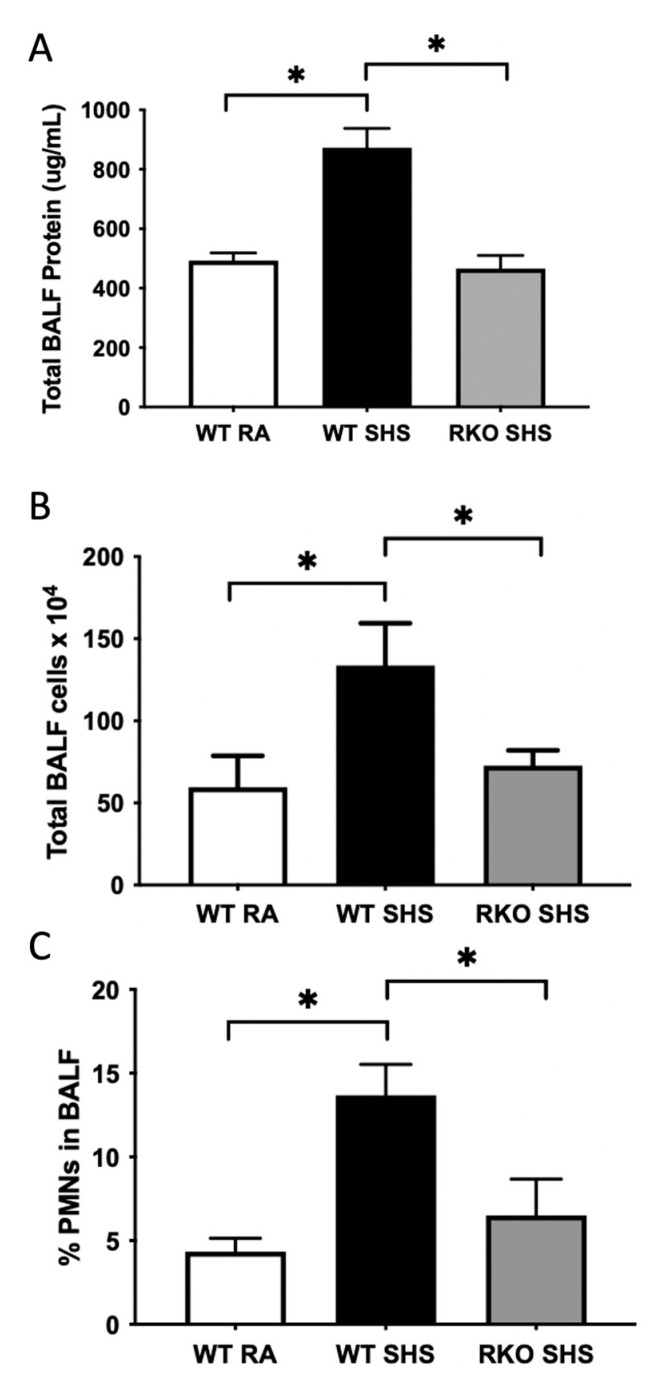
RAGE signaling related to SHS-mediated bronchoalveolar lavage fluid (BALF) alterations. (**A**), BCA assays were used to quantify total BALF protein. BALF protein level was more significantly elevated in WT + SHS-exposed mice when compared to that of WT + RA controls. BALF protein level was markedly decreased in RKO + SHS when compared to that of WT + SHS. (**B**), Number of total BALF cells was significantly increased in unexposed RAGE TG mice compared to that of WT mice (*p* = 0.03). (**B**), Total leukocyte cellularity level in BALF was also more significantly increased in WT + SHS-exposed mice when compared to that of RA controls, and there was no SHS-induced increase in BALF cellularity when comparing WT + SHS and RKO + SHS-exposed mice. (**C**), PMNs were significantly increased in WT + SHS compared to WT mice serving as RA controls. We discovered SHS-induced PMN extravasation into BALF was not elevated as was observed in WT + SHS-exposed mice. All BALF analyses listed here were conducted in BALF samples procured from at least 6 mice per group, and statistically different values are noted as * *p* ≤ 0.05. There was no difference in these three BALF assessments when comparing WT + RA and RKO + RA (not shown) controls.

We discovered evidence of pulmonary inflammation in the mice exposed to chronic SHS. The total BALF protein (an indirect assessment of elevated vascular permeability, Figure 3A) and cellularity levels (Figure 3B) were more significantly increased in the WT + SHS-exposed mice when compared to those of the WT + RA controls. The percentage of extravasated polymorphonuclear cells (PMNs) was also more significantly elevated in the WT + SHS-exposed mice compared to that of the WT + RA controls (Figure 3C). The RKO mice exposed to SHS for 6 months experienced significant attenuation of these three BALF assays; namely, SHS exposure resulted in significantly lower levels of total protein, total leukocyte cellularity, and a lower percentage of PMNs in the absence of RAGE (Figure 3A–C). 

**Figure 4:**
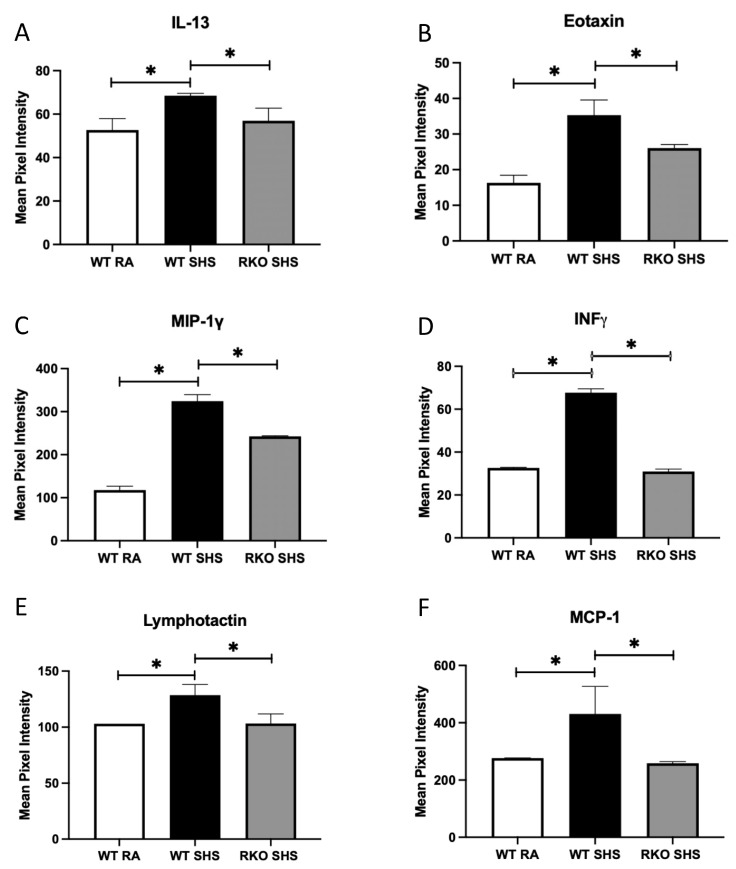
RAGE signaling related to SHS-mediated cytokine/chemokine secretion. Notable inflammatory mediators were screened in pooled BALF samples from each group. (**A**–**F**), We discovered significantly more IL-13, Eotaxin, MIP-1γ, IFN-γ, Lymphotaxin, and MCP-1 in BALF from WT + SHS-exposed mice when compared to BALF samples procured from WT + RA mice. These same chemical mediators were more significantly decreased in RKO + SHS-exposed mice compared to those of WT + SHS counterparts. Significant differences when comparing concentrations of BALF cytokine/chemokine abundance are noted as * *p* ≤ 0.05. There was no significant difference in the levels of secreted BALF mediators when comparing WT + RA and RKO + RA (not shown) controls.

**Figure 5:**
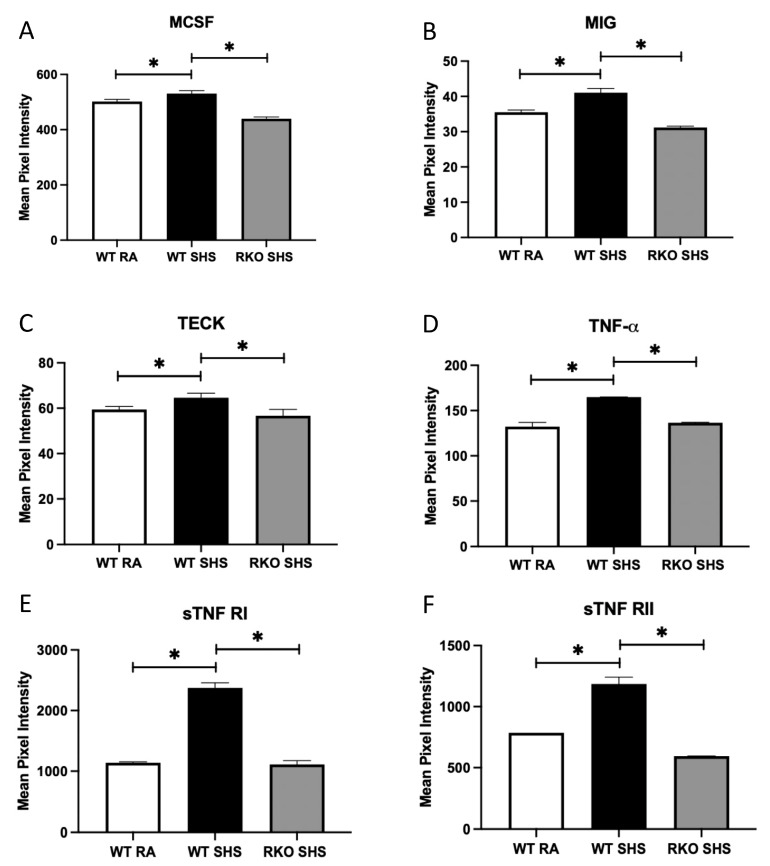
RAGE signaling related to SHS-mediated cytokine/chemokine secretion. Notable inflammatory mediators were screened in pooled BALF samples from each group. (**A**–**F**), We discovered significantly more MCSF, MIG, TECK, TNF-α, sTNFRI, and sTNFRII in BALF from WT + SHS-exposed mice when compared to BALF samples procured from WT + RA mice. The levels of these same chemical mediators were more significantly decreased in RKO + SHS-exposed mice compared to those of WT + SHS counterparts. Significant differences when comparing concentrations of BALF cytokine/chemokine abundance are noted as * *p* ≤ 0.05. There was no significant difference in the levels of secreted BALF mediators when comparing WT + RA and RKO + RA (not shown) controls.

The analysis of a mouse inflammation antibody array determined that the BALF contained increased quantities of numerous pro-inflammatory cytokines/chemokines. In particular, we observed the marked secretion of IL-13, eotaxin, MIP-1γ, IFN-γ, lymphotactin, MCP-1, MCSF, MIG, TECK, and TNF family members, including TNF-α, sTNF-R1 and sTNF-R2, in the WT + SHS-exposed animals compared to that of the WT + RA controls (Figure 4A–F and Figure 5A–F). In each instance, 6 months of chronic SHS exposure resulted in significantly more secretion of these mediators at the conclusion of the time course. Further, the levels of each of these molecular mediators were more significantly decreased in the RKO + SHS-exposed mice compared to those of the WT + SHS-exposed mice (Figure 4A–F and Figure 5A–F). 

## 3. Discussion

The pulmonary apparatus is positioned as the interface between the organism and its surrounding environment. Accordingly, exposure to deleterious stimuli, including first and SHS, can be detected in exposed lung tissue, which may then initiate cascades that orchestrate cellular responses. We and many others have previously shown that the physiological responses can seemingly be immediate. For instance, acute alterations in activated genetic programs and the secretion of tissue remodeling molecules can be almost immediately observed, often during the intervening hours or days. During such an inflammatory response, the injured tissues specifically release pro-inflammatory cytokines and chemokines to attract white blood cells to the damaged area. Local vascular endothelial cells become more permeable as leukocytes extravasate into the site of the injury. Increased vascular permeability further allows other proteins such as cytokines and chemokines to percolate across endothelial barriers into the surrounding tissue [34]. In a chronically inflamed lung, leukocyte extravasation and the results of their activation could be detected in the highly vascularized parenchyma by examining elaborated proteins and cells in distal lung airspaces. Our discovery of a likely role for RAGE in the processes involved during chronic inflammation, including leukocytic recruitment, has been discussed by others [17,35,36]. Furthermore, our observation that RAGE influences SHS-induced leukocyte diapedesis in the lungs via activated chemotactic signaling portends that a robust inflammatory event is underway following chronic exposure. 

We observed elevated Ras and NF-kB activation in the chronically exposed mice lungs. Research by others has shown that Ras is a key molecular switch implicated in the inflammatory foci of exposed lungs with relevance to both neoplastic and non-neoplastic responses [37]. In fact, de novo Ras activation is a common intracellular switch that leads to not only inflammatory states such as COPD, but other lung pathologies including fibrosis and cancer as well [37,38,39]. As reviewed in a recent publication by Sharma et al., the connections between RAGE, Ras, NF-kB, and general inflammation are increasingly becoming clarified [40]. For instance, this work and others describe that the perpetuation of inflammatory lung diseases may be modeled via RAGE expression and the availability of soluble RAGE (sRAGE) as a decoy receptor. Furthermore, RAGE and the synthesis of RAGE axis products are highly probable to be valuable COPD biomarkers [40]. Our work adds to this developing narrative due to the discovery that chronic SHS exposure does not necessarily lead to elevated AKT signaling; rather ERK is a preferred intermediate for pro-inflammatory signaling. Abundant published studies support a clear role for pERK in inflammation, including lung inflammation observed in COPD patients [41,42]. 

The most notable contribution this work makes to the evolving understanding of inflammation following chronic SHS exposure is the cytokine/chemokine signatures detected in BALF from animals that can (WT) or cannot (RKO) express RAGE. Recent research suggests that the quantification of cytokines has a significant value in clinical medicine and provides insights into physiological processes [43]. While we did not assess whether cytokines were clinically significant, we identified 12 unique molecules that were each significantly elevated in the WT animals capable of RAGE signaling; these same molecules were each significantly downregulated in the RKO animals following SHS exposure. Interleukin-13 (IL-13) is a secreted immunoregulatory cytokine that is linked to inflammatory reactions and cancer pathogenesis. IL-13 differentially impacts inflammatory cytokine production and promotes immunoglobulin class switching on B cells. Recent research suggested that IL-13 is a key potentiator of airway inflammation, remodeling, and lung function decline [44]. Interestingly, IL-13 also cooperatively regulates IFN-γ synthesis (as we have also observed here), and in relation to the induction of airway disease, it can induce a class of protein-degrading enzymes known as matrix metalloproteases (MMPs) [45,46]. We also discovered that eotaxin was impacted, which is another mediator induced by IL-13 in the alveolar epithelial cells [47]. Eotaxin is a leukocyte chemoattractant, as are other key leukocyte chemoattractants uncovered by our cytokine/chemokine screening, including MIP-1γ, lymphotactin, MCP-1, MIG, TECK, and TNF-α. Of note, MIG (CXCL9) is modulated by IFN-g and is highly chemotactic for leukocytes, as was recently revealed in models of COPD [48]. Like MIG, a host of chemoattractive markers involved in macrophage recruitment and activation were detected, including MIP-1γ, lymphotactin, MCP-1, TECK, and TNF-α. In particular, MIP-1γ, MCP-1 [49], and GMSF function in either the activation of macrophages or during the differentiation of myeloid progenitors. Of particular interest, MCSF is produced by macrophages, fibroblasts, mast cells, and lymphocytes during inflammation or in response to multiple other cytokines. It functions during the proliferation and differentiation of myeloid cells into eosinophils, monocytes/macrophages, and neutrophils, as we observed during SHS exposure.

A key pathway discovered by our cytokine/chemokine array centered on the biology of TNF following chronic SHS exposure. TNF-α is a ligand that binds to TNFRSF1A/TNFR1 and TNFRSF1B/TNFBR, and it perpetuates signaling central to inflammation, apoptosis, proliferation, invasion, angiogenesis, metastasis, and morphogenesis. It is mainly secreted by macrophages and damaged epithelial cells; inflammatory pathways are specifically modulated when in their secreted form. We discovered the differential expression of both TNF-α as well as its two main soluble receptor types (sTNFRI and sTNFRII), which dephosphorylate the membrane form of TNF when engaged [50,51]. 

Collectively, these data demonstrate a plausible role for RAGE in the coordination of pulmonary inflammatory responses that result from chronic SHS exposure. The experimental outcomes are compelling; however, limitations should be addressed in future pursuits that seek to decipher mechanistic roles for RAGE over the entire time course of exposure. For example, these convincing outcomes demonstrate a likely role for RAGE, but these results only represent a static measurement of inflammatory metrics after a chronic exposure program. Additional research would be particularly telling should such include assays throughout the chronic time course. Furthermore, subsequent endeavors should also assess the degree to which other stimuli similarly employ RAGE signaling. Such studies could examine the effects of other COPD risk factors in non-smokers, such as biomass exposure, occupational exposure, and/or air pollutants, such as diesel particulate matter, in the context of RAGE availability. In the end, determining the extent of RAGE involvement in pulmonary inflammatory responses could contribute to the elucidation of potential therapeutic measures that ameliorate pulmonary disease exacerbations. 

## 4. Methods and Materials

### 4.1. Animals and Exposure to SHS

Animals were housed in a standard pathogen-free facility on a 12 h light cycle and given access to food and water ad libitum. Adolescent mice that were 40 days old (post-natal day 40, PN40) [52,53] were exposed to secondhand smoke (SHS) or room air (RA) for a period of 6 months (approximately 24 weeks). Wild-type (WT) and RAGE knockout (RKO) mice exposed to SHS (n = 6 mice per group) were restrained and delivered SHS via a nose-only delivery tower (InExpose System, Scireq, Montreal, QC, Canada) as previously detailed [31]. The mice were exposed for up to five days weekly in accordance with pilot studies and current literature regarding the time required to elicit physiological responses. The mice exposed to RA were similarly restrained and allowed to breathe room air (n = 6 mice per group). There were no deaths observed during the course of the study. The animals from all groups were sacrificed upon completion of this 6-month time course. The right lung was tied off with suture string, and the left lung was lavaged to collect bronchoalveolar lavage fluid (BALF). The unlavaged right lobes were resected and immediately snap-frozen in liquid nitrogen for the analysis of markers in total lung lysates. Animal use protocols were approved by the Institutional Animal Care and Use Committee (IACUC) at Brigham Young University and carried out in accordance with the prevailing regulations. The IACUC protocol number was 21-0203, and the approval dates were from 17 March 2021 to 16 March 2024.

### 4.2. Ras and NF-kB Analyses

As detailed previously [32,54], active Ras in the total lung lysates was detected using the Ras Activation ELISA kit (Cat # 17-218; Millipore, Burlington, MA, USA) and compared to the positive controls included in the kit according to the manufacturer’s instructions. A calorimetric high-throughput FACE assay (Cat # 42396; Active Motif, Carlsbad, CA, USA) was employed to quantify NF-kB via the specific detection of active p65 NF-kB in the total lung lysates.

### 4.3. Immunoblot Analyses

Snap-frozen lung samples were homogenized in RIPA protein lysis buffer supplemented with a cocktail of protease and phosphatase inhibitors (Thermo Fisher Scientific, Pittsburg, PA, USA). The total protein in lung lysates was quantified with a BCA Protein Assay kit (Thermo Fisher Scientific). Immunoblotting using anti-phospho AKT (Cat # 4060; 1:250, Cell Signaling Technology, Danvers, MA, USA), anti-phospho ERK (Cat # 4370; 1:500, Cell Signaling), or anti-Beta actin (Cat # 4970; 1:5000, Cell Signaling) was performed using standard procedures. Protein detection was facilitated via exposure to fluorescent secondary antibodies (680RD Donkey anti-Rabbit or 800CW Donkey anti-Rabbit at 1:2500; from LI-COR, Lincoln, NE, USA). Fluorescent signals were captured using the Odyssey DLx Near-Infrared Fluorescence Imaging System (LI-COR), and then quantitatively analyzed using Image Studio Lite (LI-COR). At least six mice per group were included in each immunoblot. 

### 4.4. Bronchoalveolar Lavage Fluid (BALF) Analyses

On the date of sacrifice, the left lobes were isolated, and a 20-guage cannula was inserted into the trachea. PBS was used to lavage the lobes four times, which were then extracted. The resulting bronchoalveolar lavage fluid (BALF) was collected and centrifuged at 4 °C; the supernatant was removed and preserved for subsequent inflammatory array analysis as described below. Following centrifugation, the pellet was resuspended in 500 µL of sterile PBS; the total cell counts were measured manually using a hemocytometer, where counts were performed at least three times and averaged. A portion of the resuspension was fixed on a histological slide via cytospin for differential staining. The percentage of polymorphonuclear (PMN) cells was determined by counting the PMNs among 200 total cells in each differentially stained slide. Counts were repeated at least three times and averaged. From six to eight mice were used per treatment group for each BALF assessment. 

### 4.5. Inflammatory Molecule Analyses

The total protein in BALF supernatants was measured using a BCA Protein Assay Kit (Thermo Fisher Scientific), and 25 µg of protein from six animals in each treatment group was collected to create a sample pool with a final concentration of 150 µg/mL. Sample pools were added to membranes from a mouse inflammation antibody array (Cat # ab13399; Abcam, Waltham, MA, USA) containing specific capture antibodies and allowed to incubate overnight before being retrieved and incubated again with a second antibody array membrane. Biotinylated antibodies were then added to each membrane and incubated overnight followed by a final incubation with a streptavidin-conjugated fluorescent label to detect the cytokine expression. Membranes were imaged using the fluorescence imaging system previously mentioned (LI-COR), and then quantified using Image J (U.S. National Institutes of Health, Bethesda, MD, USA) [55]. Signal intensities were compared to positive controls included on each membrane. 

### 4.6. Statistical Analysis

GraphPad Prism software (GraphPad version 10.1.0; Santa Clara, CA, USA) was used for statistical analyses. Mean values ± S.D. per animal group were assessed by one- and two-way analysis of variance (ANOVA), while considering normal variances and variables. Mann–Whitney tests were used for immunoblotting analysis. The results are representative, and those with *p* values < 0.05 were considered significant.

## Data Availability

All data are presented within the article. Data and other materials are available from the corresponding author on reasonable request.
